# SP Retroperitoneal Robotic Nephrectomy via Lower Anterior Approach for Xanthogranulomatous Pyelonephritis in a Horseshoe Kidney

**DOI:** 10.1590/S1677-5538.IBJU.2025.0383

**Published:** 2025-08-05

**Authors:** William A. Langbo, Arianna Biasatti, Carol L. Feng, Taylor Stivali, Ruben Mercado Santos, Sameer Ansari, Riccardo Autorino

**Affiliations:** 1 Rush University Medical Center Department of Urology Chicago IL USA Department of Urology, Rush University Medical Center, Chicago, IL, USA

## Abstract

**Purpose:**

Xanthogranulomatous pyelonephritis (XGP) represents an uncommon and challenging clinical scenario (1). XGP in a horseshoe kidney poses additional surgical complexity due to the anatomical anomaly (2). Robot-assisted radical nephrectomy (RARN) for XGP has been rarely reported (3), and single-port (SP) RARN via a retroperitoneal lower anterior approach (LAA) represents a recent innovation in the field (4). The aim of this video is to describe a case of SP RARN via LAA in a horseshoe kidney with XGP.

**Materials and Methods:**

We report the case of a 51-year-old female (BMI 23 kg/m^2^) with XGP in a horseshoe kidney. A CT scan revealed severe left renal enlargement and hydroureteronephrosis, and a renal scan demonstrated <5% function of the left kidney, with a normal right kidney. The decision was made to proceed with SP RARN (5). Briefly, the patient was positioned in a modified supine position, and LAA was obtained via a 6-cm incision placed two fingerbreadths medial to the anterior superior iliac spine. The retroperitoneal space was bluntly developed, the large access port kit was introduced, and a 15-mm assistant port was placed through the same skin incision but via a separate fascial incision. The left renal hilum was identified, and the renal artery, vein, and ureter were ligated and transected. The isthmus was controlled using bipolar electrocautery and sharply divided. The kidney was freed and extracted.

**Results:**

Total operative time was 4.5 hours, estimated blood loss was 150 mL, and no perioperative complications occurred. The patient tolerated the procedure well and was discharged on postoperative day 1. The specimen weighed 500 g and showed a multinodular kidney with pathology consistent with XGP. The patient remained asymptomatic and complaint-free at the 3-month follow-up.

**Conclusions:**

SP RARN for XGP is feasible and can be safely performed. The LAA provides a versatile surgical approach and facilitates rapid postoperative recovery.

Available at: VIDEO

## Data Availability

Not applicable
